# Validation of SinoSCORE for isolated CABG operation in East China

**DOI:** 10.1038/s41598-017-16925-x

**Published:** 2017-12-01

**Authors:** Xiue Ma, Yunqian Wang, Lingtong Shan, Zhengqiang Cang, Chang Gu, Nianyi Qu, Qifan Li, Jun Li, Zhenhua Wang, Yangyang Zhang

**Affiliations:** 1 Research Center for Translational Medicine, East Hospital, Tongji University School of Medicine, Shanghai, China; 2 Key Laboratory of Arrhythmias of the Ministry of Education of China, East Hospital, Tongji University School of Medicine, Shanghai, China; 3 The First Clinical Medical College of Nanjing Medical University, Nanjing, China; 4 Department of Thoracic Surgery, Shanghai Chest Hospital, Shanghai Jiao Tong University, Shanghai, China; 5 College of information technology, Shanghai Ocean University, Shanghai, China; 6 Department of Cardiovascular Surgery, East Hospital, Tongji University School of Medicine, Shanghai, 200120 China

## Abstract

From January 2010 to December 2016, 1616 consecutive patients who underwent isolated coronary artery bypass grafting (CABG) were evaluated for their predicted mortality according to the online Sino System for Coronary Operative Risk Evaluation (SinoSCORE), European System for Cardiac Operative Risk Evaluation II (EuroSCORE II) and Society of Thoracic Surgeons (STS) risk evaluation system. The calibration and discrimination in the total and in the subsets were assessed by the Hosmer-Lemeshow (H-L) statistics and by the C statistics respectively, to evaluate the efficiency of the three risk evaluation systems. The realized mortality was 1.92% (31/1616). The predictive mortality of SinoSCORE, EuroSCORE II and STS risk evaluation system were 1.35%, 1.74% and 1.05%, respectively. SinoSCORE achieved best discrimination. When grouping by risk, SinoSCORE also achieved the best discrimination in high-risk group, followed by STS risk evaluation system and EuroSCORE II while SinoSCORE and EuroSCORE II had excellent performance in low-risk group. In terms of calibration, SinoSCORE, EuroSCORE II and STS risk evaluation system all achieved positive calibrations (H-L: P > 0.05) in the overall population and grouped subsets. SinoSCORE achieved good predictive efficiency in East China patients undergoing isolated CABG and showed no compromise when compared with EuroSCORE II and STS risk evaluation system.

## Introduction

Coronary artery disease (CAD) is a common cardiovascular disease that seriously damages human health. Due to the rapid economic development and higher incidence of CAD in developing countries, China has observed an upsurge in patients undergoing CABG over the last decade^1^. The high risk of heart surgery during perioperation has gradually come to the attention of surgeons. Several risk evaluation systems, which quantify the risk by the patients’ data and predict their mortality or morbidity, have been developed and have received positive evaluations during the last two decades worldwide. Of these systems, two have become predominant: EuroSCORE in Europe and STS risk evaluation system in North America^2^. In China, Fuwai Hospital created a national multi-center database of patients undergoing isolated CABG known as the Chinese Coronary Artery Bypass Grafting Registry Study ^3,4^. Based on the more than 9,000 patients in this database, Sino System for Coronary Operative Risk Evaluation (SinoSCORE) was published in 2010^5^.

SinoSCORE, EuroSCORE II and STS risk evaluation system were all developed using heart surgery patients in different regions and were well received, to varying degrees, for clinical application. The aim of this study is to validate SinoSCORE with isolated CABG patients in East China and compare the accuracy of predictive mortality of the three systems.

## Results

**Figure 1:**
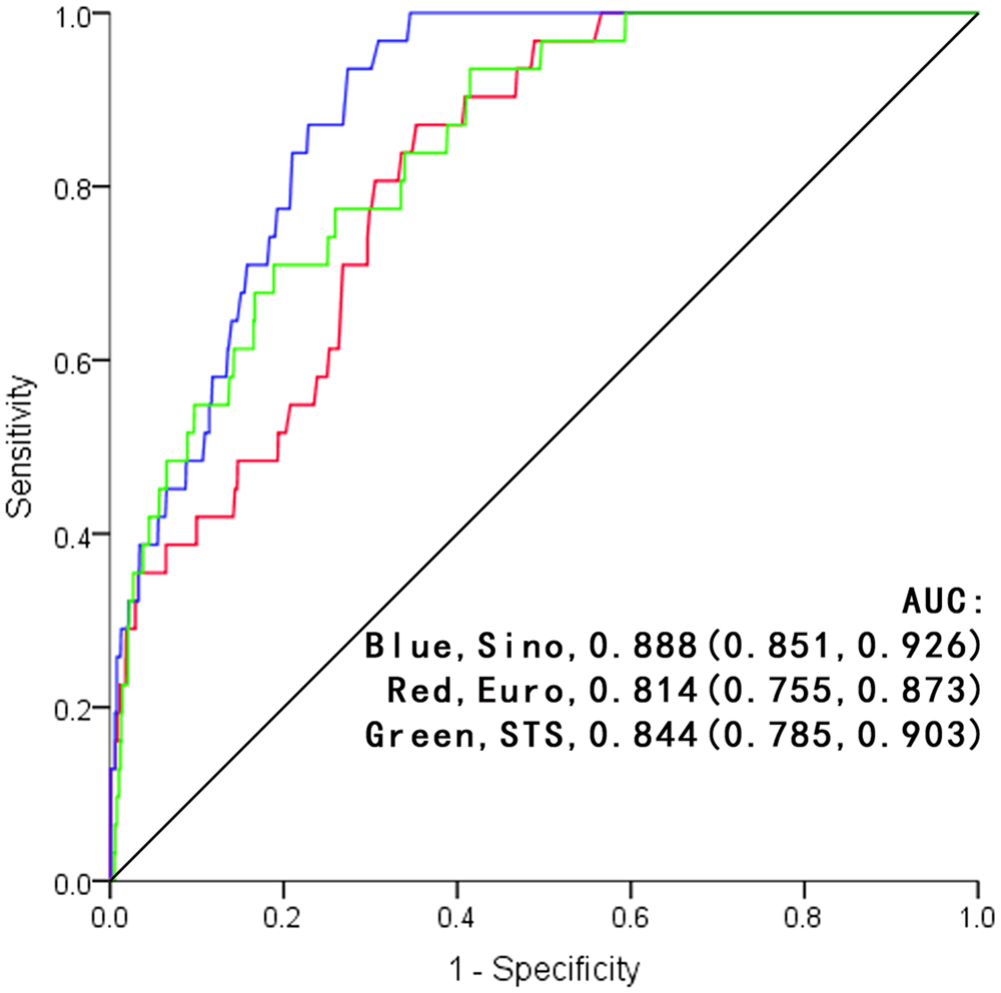
The receiver operating characteristic curves of three risk evaluation systems in total patients For all of total patients, the receiver operating characteristic curves of EuroCORE II was 0.814, of SinoSCORE was 0.888, and of STS risk evaluation system was 0.844, respectively.

**Figure 2:**
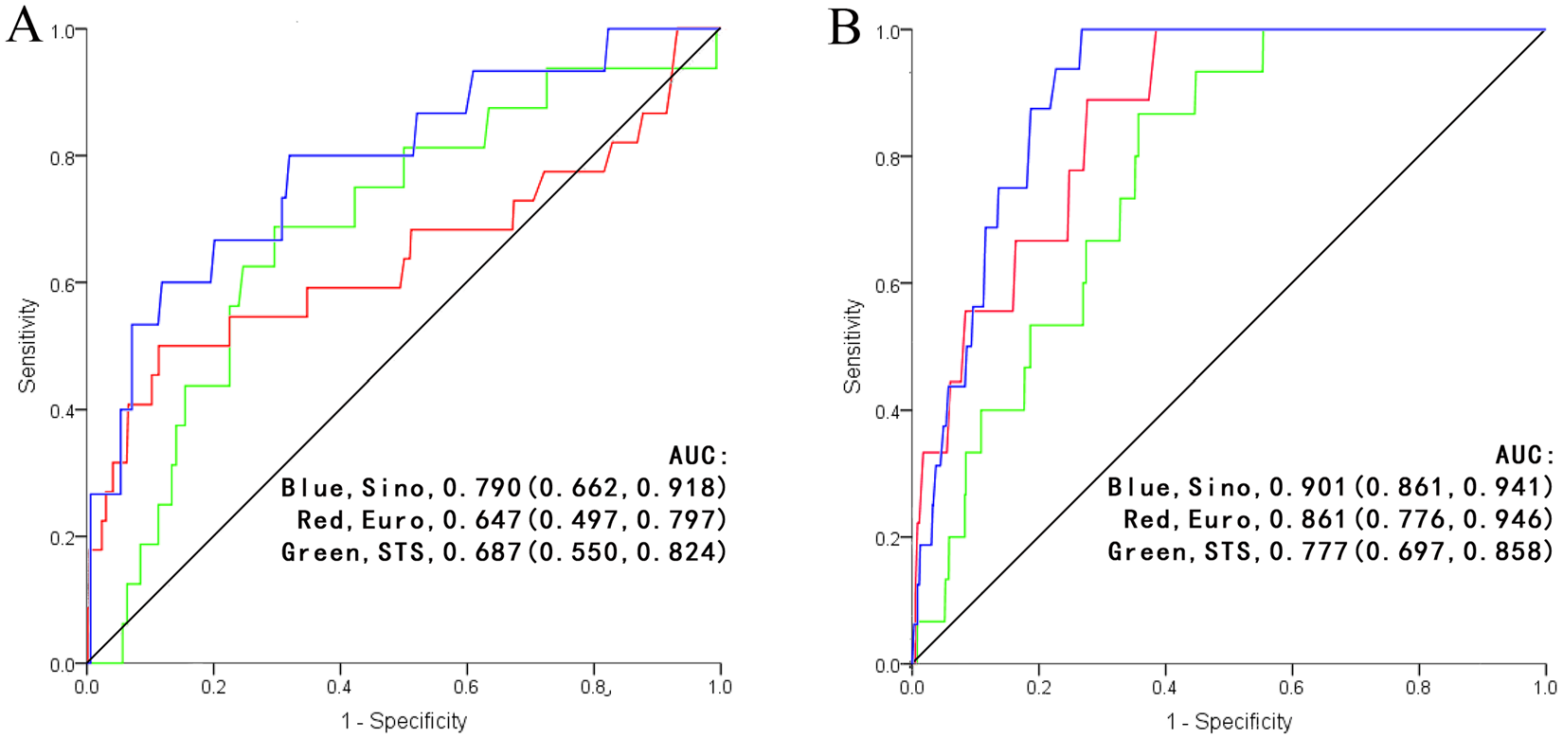
The receiver operating characteristic curves of the three risk evaluation systems with subsets. (**A**) The receiver operating characteristic curves of the three risk evaluation systems with high risk (EuroCORE II 0.647, SinoSCORE 0.790 and STS risk evaluation system 0.687). (**B**) The receiver operating characteristic curves of the three risk evaluation systems with low risk (EuroCORE II 0.861, SinoSCORE 0.901 and STS risk evaluation system 0.777).

For all 1616 patients in study, the realized mortality was 31 patients, or 1.92%. The baseline clinical characteristics of total patients were summarised in Table 1. The baseline data of subsets grouped by risk were shown in Table 2 and Table 3. The realized and predictive mortality rates for total patients and subsets were shown in Table 4. The predictive mortality of EuroSCORE II was1.74 ± 1.37% (95%CI 1.67–1.81), while SinoSCORE was 1.35 ± 3.30% (95%CI 1.19–1.51) and STS risk evaluation system was 1.05 ± 1.45% (95%CI 0.98–1.12). The receiver operating characteristic (ROC) curves of the three systems for total patients and subgroups were shown in Fig. 1 and Fig. 2. SinoSCORE achieved excellent discrimination (AUC = 0.888), followed by STS risk evaluation system (AUC = 0.844) and EuroSCORE II (AUC = 0.814). When grouping by risk, SinoSCORE (AUC = 0.790) also achieved the best discrimination in high-risk group, followed by STS risk evaluation system (AUC = 0.681) and EuroSCORE II (AUC = 0.647), while SinoSCORE (AUC = 0.901) and EuroSCORE II (AUC = 0.861) had excellent performance in low-risk group (Fig. 2, Table 4).

**Table 1:** CABG patient baseline clinical characteristics.

Risk factors	Total (N = 1616)
Age (y)	65.21 ± 8.50(35–85)
Female (n, %)	349(21.60)
Weight (kg)	69.06 ± 10.40(37–125)
Height (cm)	166.84 ± 6.99(144–185)
BMI (kg/m^2^)	24.76 ± 3.08(15.60–39.06)
Morbid obesity (n, %)	82(5.07)
Body surface area (m^2^)	1.75 ± 0.16(1.26–2.55)
Diabetes (n, %)	518(32.05)
Hypertension (n, %)	1087(67.26)
Renal failure (n, %)	21(1.30)
Serum creatinine (μmol/l)	82.74 ± 41.94(29.20–937.00)
Ccr (ml/min)	81.40 ± 27.07(6.53–246.55)
Cerebrovascular accident (n, %)	40(2.48)
COPD (n, %)	44(2.72)
Peripheral vascular disease (n, %)	40(2.48)
Cardiovascular surgery (n, %)	79(4.89)
Atrial flutter and fibrillation (n, %)	42(2.60)
Accompanied by pulmonary hypertension (n, %)	157(9.72)
Myocardial infarction (n, %)	213(13.18)
Unstable angina pectoris (n, %)	850(52.60)
Number of diseased coronary vessels (n)	2.85 ± 0.46(1–3)
Three-vessel coronary disease (n, %)	1448(89.60)
NYHA IV (n, %)	34(2.10)
LVEF (%)	61.13 ± 7.44(20.90–75.00)
Severe preoperative status (n, %)	78(4.83)
Preoperative IABP (n, %)	12(0.74)
Status of surgery	
Elective (n, %)	1548(95.79)
Urgent (n, %)	51(3.16)
Salvage (n, %)	17(1.05)
Number of grafts (n)	3.53 ± 1.07(1–8)
Hospital mortality (n, %)	31(1.92)

Abbreviations: BMI, body mass index; COPD, chronic obstructive pulmonary disease; Scr, Serum creatinine; Ccr, endogenous creatinine clearance rate; LVEF, left ventricular ejection fraction.

**Table 2:** Baseline clinical characteristics of low risk groups.

Risk factors	SinoLRG	EuroLRG	STSLRG	*P*
Number	1432	1133	1458	
Age (y)	64.65 ± 8.29(38–85)	62.56 ± 7.71 (35–81)	64.33 ± 8.18(35–85)	0.000
Female (n, %)	300(20.95)	195(17.21)	291(19.96)	0.053
Weight (kg)	69.49 ± 10.36(37–125)	70.78 ± 10.10(37–125)	69.75 ± 10.26(37–125)	0.004
Height (cm)	167.07 ± 6.92(144–185)	167.87 ± 6.58(148–184)	167.26 ± 6.76(144–184)	0.009
BMI (kg/m^2^)	24.85 ± 3.09(15.60–39.06)	25.08 ± 3.05(15.60–39.06)	24.89 ± 3.05(15.60–39.06)	0.128
Morbid obesity (n, %)	76(5.31)	65(5.74)	77(5.28)	0.811
Body surface area (m^2^)	1.76 ± 0.16(1.26–2.55)	1.78 ± 0.15(1.26–2.55)	1.76 ± 0.16(1.26–2.55)	0.002
Diabetes (n, %)	448(31.28)	322(28.42)	457(31.34)	0.204
Hypertension (n, %)	953(66.55)	735(64.87)	971(66.60)	0.591
Renal failure (n, %)	11(0.77)	7(0.62)	10(0.69)	0.901
Serum creatinine (μmol/l)	80.30 ± 38.21(29.20–937.00)	78.66 ± 39.11(29.20–937.00)	80.63 ± 37.70(29.20–937.00)	0.391
Ccr (ml/min)	83.62 ± 26.28(6.53–246.55)	89.09 ± 25.39(6.53–246.55)	83.97 ± 26.21(6.53–246.55)	0.000
Cerebrovascular accident (n, %)	34(2.37)	18(1.59)	32(2.19)	0.002
COPD (n, %)	28(1.96)	16(1.41)	37(2.54)	0.127
Peripheral vascular disease (n, %)	35(2.44)	15(1.32)	32(2.19)	0.119
Previous cardiac surgery (n, %)	50(3.49)	17(1.50)	70(4.80)	0.000
Atrial flutter and fibrillation (n, %)	38(2.65)	28(2.47)	37(2.54)	0.957
Pulmonary hypertension (n, %)	138(9.64)	112(9.89)	140(9.60)	0.967
Myocardial infarction (n, %)	170(11.87)	131(11.56)	182(12.48)	0.760
Unstable angina pectoris (n, %)	754(52.65)	563(49.69)	756(51.85)	0.300
Number of diseased coronary vessels (n)	2.85 ± 0.46(1–3)	2.83 ± 0.50(1–3)	2.85 ± 0.47(1–3)	0.491
Three-vessel coronary disease (n, %)	1283(89.59)	1003(88.53)	1302(89.30)	0.647
NYHA IV (n, %)	20(1.40)	8(0.71)	23(1.58)	0.017
LVEF (%)	61.53 ± 6.98(20.90–75.00)	61.47 ± 7.04(20.90–74.90)	61.29 ± 7.20(20.90 ± 75.00)	0.641
Severe preoperative status (n, %)	52(3.63)	48(4.24)	64(4.39)	0.558
Preoperative IABP (n, %)	6(0.42)	4(0.35)	7(0.48)	0.967
Status of surgery				0.453
Elective (n, %)	1388(96.93)	1090(96.20)	1402(96.16)	
Urgent (n, %)	43(3.00)	35(3.09)	46(3.16)	
Salvage (n, %)	1(0.07)	8(0.71)	10(0.69)	
Number of grafts (n)	3.56 ± 1.09(1–8)	3.59 ± 1.13(1–8)	3.56 ± 1.08(1–8)	0.726
Hospital mortality (n, %)	16(1.12)	9(0.79)	15(1.03)	0.705

Abbreviations: BMI, body mass index; COPD, chronic obstructive pulmonary disease; Scr, Serum creatinine; Ccr, endogenous creatinine clearance rate; LVEF, left ventricular ejection fraction; SinoLRG, low risk group of SinoSCORE; EuroLRG, low risk group of EuroSCORE; STSLRG, low risk group of STS.

**Table 3:** Baseline clinical characteristics of high risk groups.

Risk factors	SinoHRG	EuroHRG	STSHRG	*P*
Number	184	483	158	
Age (y)	69.58 ± 8.85(35–85)	71.43 ± 6.85(45–85)	73.37 ± 6.83(48–85)	0.000
Female (n, %)	49(26.63)	154(31.88)	58(36.71)	0.134
Weight (kg)	65.78 ± 10.13(40–95)	65.03 ± 9.98(40–101)	62.72 ± 9.49(43–95)	0.006
Height (cm)	165.05 ± 7.24(150–181)	164.43 ± 7.31(144–185)	163.02 ± 7.82(148–185)	0.034
BMI (kg/m^2^)	24.08 ± 2.94(16.65–33.20)	24.01 ± 3.02(16.61–36.89)	23.59 ± 3.09(17.58–32.45)	0.256
Morbid obesity (n, %)	6(3.26)	17(3.52)	5(3.16)	0.979
Body surface area (m^2^)	1.70 ± 0.16(1.30–2.15)	1.68 ± 0.16(1.30–2.23)	1.64 ± 0.15(1.31–2.15)	0.007
Diabetes (n, %)	70(38.04)	196(40.58)	61(38.61)	0.801
Hypertension (n, %)	134(72.83)	352(72.88)	116(73.42)	0.990
Renal failure (n,%)	10(5.43)	14(2.90)	11(6.96)	0.059
Serum creatinine (μmol/l)	101.68 ± 60.80(41.60–602.00)	92.31 ± 46.58(39.90–602.00)	102.18 ± 66.97(42.00–602.00)	0.263
Ccr (ml/min)	64.13 ± 27.01(9.38–154.81)	63.35 ± 21.82(9.38–184.16)	57.64 ± 23.15(9.38–112.85)	0.023
Cerebrovascular accident (n, %)	6(3.26)	22(4.55)	8(5.06)	0.020
COPD (n, %)	16(8.70)	28(5.80)	7(4.43)	0.228
Peripheral vascular disease (n, %)	5(2.72)	25(5.18)	8(5.06)	0.382
Previous cardiac surgery (n, %)	29(15.76)	62(12.84)	9(5.70)	0.013
Atrial flutter and fibrillation (n, %)	4(2.17)	14(2.90)	5(3.16)	0.835
Pulmonary hypertension (n, %)	19(10.33)	45(9.32)	17(10.76)	0.840
Myocardial infarction (n, %)	43(23.37)	82(16.98)	31(19.62)	0.164
Unstable angina pectoris (n, %)	95(51.63)	287(59.42)	94(59.49)	0.546
Number of diseased coronary vessels (n)	2.86 ± 0.43(1–3)	2.91 ± 0.34(1–3)	2.91 ± 0.33(1–3)	0.395
Three-vessel coronary disease (n, %)	164(89.13)	445(92.13)	146(92.41)	0.471
NYHA IV (n, %)	14(7.61)	26(5.38)	11(6.96)	0.571
LVEF (%)	57.95 ± 9.80(30.60–71.90)	60.32 ± 8.27(30.60–75.00)	59.58 ± 9.28(30.60–70.40)	0.036
Severe preoperative status (n, %)	26(14.13)	30(6.21)	14(8.86)	0.005
Preoperative IABP (n, %)	6(3.26)	8(1.66)	5(3.16)	0.049
Status of surgery				0.002
Elective (n, %)	160(86.96)	458(94.82)	146(92.41)	
Urgent (n, %)	8(4.35)	16(3.31)	5(3.16)	
Salvage (n, %)	16(8.70)	9(1.86)	7(4.43)	
Number of grafts (n)	3.26 ± 0.83(1–5)	3.38 ± 0.89(1–6)	3.24 ± 0.84(1–5)	0.112
Hospital mortality (n, %)	15(8.15)	22(4.55)	16(10.13)	0.026

Abbreviations: BMI, body mass index; COPD, chronic obstructive pulmonary disease; Scr, Serum creatinine; Ccr, endogenous creatinine clearance rate; LVEF, left ventricular ejection fraction; SinoHRG, high risk group of SinoSCORE; EuroHRG, high risk group of EuroSCORE; STSHRG, high risk group of STS.

**Table 4:** Realized and predictive mortality rates of the three systems.

	Number of patients	Deaths	Realized mortality(%)	Predictive mortality(%,95%CI)	AUC(value,95%CI)	H-L statistics
SinoHRG	184	15	8.15	5.58 ± 8.64(4.33–6.84)	0.790(0.662–0.918)	0.988
SinoLRG	1432	16	1.12	0.81 ± 0.37(0.79–0.83)	0.901(0.861–0.941)	0.994
T Sino	1616	31	1.92	1.35 ± 3.30(1.19–1.51)	0.888(0.851–0.926)	0.408
EuroHRG	483	22	4.55	3.13 ± 1.78(2.97–3.29)	0.647(0.497–0.797)	0.103
EuroLRG	1133	9	0.79	1.15 ± 0.37(1.13–1.17)	0.861(0.776–0.946)	1.000
T Euro	1616	31	1.92	1.74 ± 1.37(1.67–1.81)	0.814(0.755–0.873)	0.973
STSHRG	158	16	10.13	3.45 ± 3.70(2.87–4.03)	0.687(0.550–0.824)	0.898
STSLRG	1458	15	1.03	0.79 ± 0.42(0.77–0.82)	0.777(0.697–0.858)	1.000
T STS	1616	31	1.92	1.05 ± 1.45(0.98–1.12)	0.844(0.785–0.903)	0.934

Abbreviations: SinoHRG, high risk group of SinoSCORE; SinoLRG, low risk group of SinoSCORE; T Sino, total patients of SinoSCORE; EuroHRG, high risk group of EuroSCORE II; EuroLRG, low risk group of EuroSCORE II; T Euro, total patients of EuroSCORE II; STSHRG, high risk group of STS; STSLRG, low risk group of STS; T STS, total patients of STS; AUC, area under receiver operating characteristic curve; H-L statistics, Hosmer-Lemeshow statistics.

In terms of model calibration, SinoSCORE (H-L: *P* = *0*.*405*), EuroSCORE II (H-L: *P* = *0*.*973*) and STS risk evaluation system (H-L: *P* = *0*.*934*) all achieved positive calibrations (H-L: *P > 0*.*05*) in the overall population. When patients were divided into high-risk group and low-risk group, the calibration was also assessed in each group by the Hosmer-Lemeshow (H-L) statistics. In the subset of high risk, SinoSCORE (H-L: *P* = *0*.*988*), EuroSCORE II (H-L: *P* = *0*.*103*) and STS risk evaluation system (H-L: *P* = *0*.*898*) achieved good calibrations (H-L: *P > 0*.*05*); so did in low-risk group: SinoSCORE (H-L: *P* = *0*.*994*), EuroSCORE II (H-L: *P* = *1*.*000*) and STS risk evaluation system (H-L: *P* = *1*.*000*) (Table 4).

**Figure 3:**
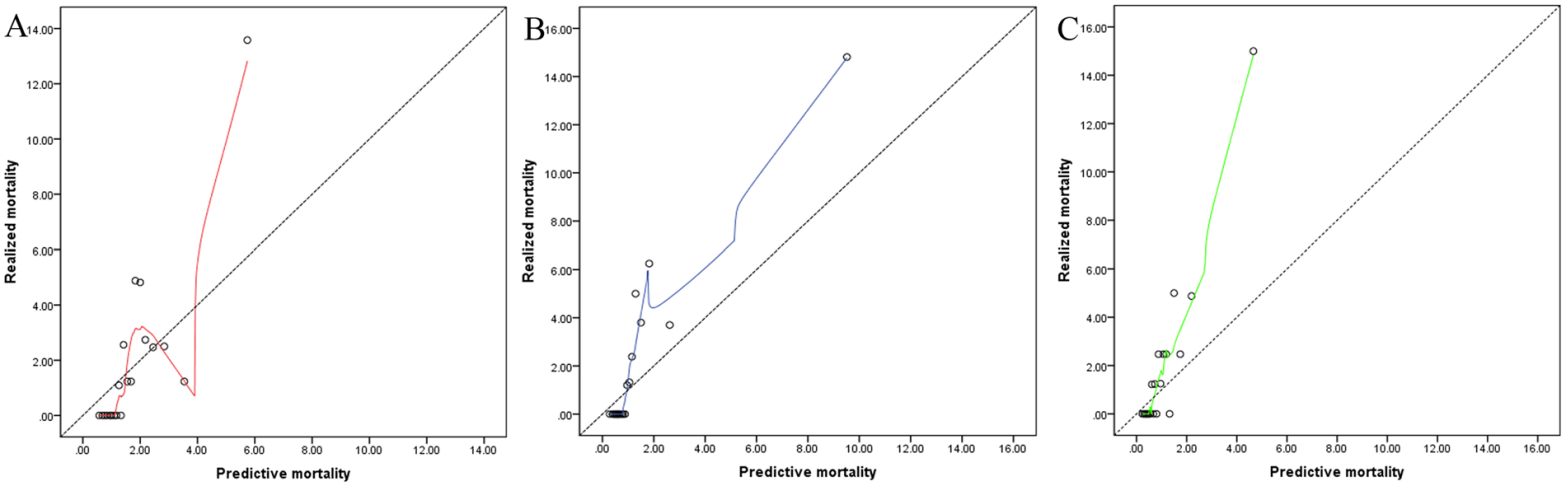
Calibration plots for the three risk evaluation systems. (**A**) Calibration plots for EuroCORE II. (**B**) Calibration plots for SinoSCORE. (**C**) Calibration plots for STS risk evaluation system.

Calibration plots showed that three risk evaluation systems deviated from the diagonal. It was explained that three risk evaluation systems underestimated mortality rates in total patients, where SinoSCORE performed slightly better than others (Fig. 3).

**Figure 4:**
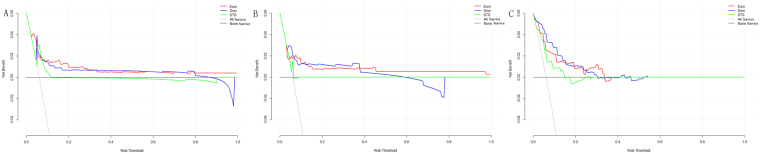
DCA showed the clinical usefulness of EuroSCORE II, SinoSCORE and STS risk evaluation system in predicting in-hospital mortality. The grey line represented the net benefit of providing surgery for all patients, assuming that all patients would survive. The black line represented the net benefit of surgery to none patients, assuming that none would survive after surgery. The red, blue and green lines represented the net benefit of applying surgery to patients according to EuroSCORE II, SinoSCORE, and STS risk evaluation system, respectively. The selected probability threshold was plotted on the abscissa. (**A**) DCA for high-risk group. (**B**) DCA for low-risk group. (**C**) DCA for entire cohort.

The decision curve analyses (DCA) represented the clinical practicability of the three risk evaluation systems to predict operative mortality. The results were showed as a graph with the selected probability threshold (i.e., the degree of certitude of postoperative mortality over which patients refused operation) plotted on the abscissa and the net benefits of the risk evaluation system on the ordinate. In the entire cohort, decision curves of EuroSCORE II and SinoSCORE were similar, and the curve of EuroSCORE II was slightly greater than the curve of SinoSCORE, included between 0 and 30%. But they were all always above the curve of STS risk evaluation system regardless of the selected threshold. (Fig. 4C) In high-risk group, the net benefits of the STS risk evaluation system were worse than those of SinoSCORE and EuroSCORE II regardless of the selected threshold. The curve of SinoSCORE was slightly greater than that of EuroSCORE II, included between 0 and 40%. (Fig. 4A) In low-risk group, the net benefit of the SinoSCORE was always greater than that of EuroSCORE II and STS risk evaluation system between 0 and 20% (Fig. 4B).

## Discussion

In recent years, because of the rapidly increasing CABG patients and the demand for high-risk surgery, both patients and surgeons have become aware of the risk evaluation system. These systems have played an important role in surgical decision-making and have improved the quality of medical treatment, preoperative patient education and consent, optimisation of the allocation of medical resources and standardisation of the comparisons among different centers or surgeons^6–8^. The risk evaluation systems were aimed at providing a more accurate assessment to guide surgery for individual patients by balancing the potential risks and benefits^9^. A thorough risk evaluation system should be established on a large database that is representative of current clinical practice, and systematic data validation should be utilised to affirm its accuracy^10^.

Risk evaluation systems for heart surgery have been under study in developed countries since decades ago, and based primarily on European (EuroSCORE II) and North American (STS risk evaluation system) databases, which may lead to the obvious errors when applied in Chinese population^11–15^. In this context, SinoSCORE, which was established with Chinese database, was developed in 2010. At the same time, the previously developed risk evaluation systems were under continuous revision to improve the accuracy and representativeness of the database due to the increasing numbers of research centers, cases, and changed or removed of outdated risk factors^16–21^. Therefore, SinoSCORE, EuroSCORE II and STS risk evaluation system were all established for several years. The first affiliated hospital of Nanjing Medical University and East hospital affiliated to Tongji University are both regional central hospitals, located in Nanjing and Shanghai, East China. Patients from the two hospitals could represent typical East China patients. Because of the vast territory of China, there are great differences in the four corners. There were some different proportions in the same risk factors between our study database and SinoSCORE database, such as age, diabetes, hypertension, renal failure, cerebrovascular accident, previous cardiac surgery and so on (Table 5). It is significant to compare the three risk evaluation systems in East China patients.

**Table 5:** The baseline risk factors of SinoSCORE database and Local database.

**Risk factors**	**SinoSCORE (N** = **9248)**	**Local (N** = **1616)**	**P**
Age (y)	62.6 ± 9.2	65.2 ± 8.5	<0.001
Female (%)	21.5	21.6	0.928
Diabetes (%)	26.4	32.1	<0.001
Hypertension (%)	63.5	67.3	0.003
Renal failure (%)	0.6	1.3	0.002
Cerebrovascular accident (%)	8.3	2.5	<0.001
COPD (%)	1.3	2.7	<0.001
Peripheral vascular disease (%)	2.5	2.5	0.957
Previous cardiac surgery (%)	2.3	4.9	<0.001
Active endocarditis (%)	0	0	1.000
Critical preoperative state (%)	4.6	4.8	0.683
Myocardial infarction (%)	9.6	13.2	<0.001
Unstable angina pectoris (%)	31.1	52.6	<0.001
Three-vessel coronary disease (%)	76.7	89.6	<0.001
Emergency (%)	7.1	4.2	<0.001
Pulmonary hypertension (%)	1.1	9.7	<0.001
LVEF 30–50% (%)	20.9	9.5	<0.001
LVEF < 30% (%)	0.9	0.1	0.001
Isolated CABG (%)	87.8	100	<0.001
Hospital mortality (%)	3.27	1.92	0.004

Abbreviations: COPD, chronic obstructive pulmonary disease; LVEF, left ventricular ejection fraction.

Validation literatures on Chinese patients excluding isolated valve surgery, only one had been published that indicated the EuroSCORE II performed well in predicting mortality in total and in the low-middle risk group, whereas not in the high-risk group^22^. Although the EuroSCORE II database had significant differences with our study database in parity of regions and populations, it achieved excellent predictive value in total (AUC = 0.814), as well as in low-risk groups of patients (AUC = 0.861). Similar to the result of Bai *et al*.^22^, the discrimination of EuroSCORE II in the high-risk group was not satisfactory. The number of patients at high-risk in EuroSCORE II was two times higher than in SinoSCORE and STS risk evaluation system, some patients with low-risk were assigned to the high-risk group, and which might be the reason contributed to the discrimination of EuroSCORE II in the high-risk group was not satisfactory.

As well-known as EuroSCORE II, STS risk evaluation system was composed of three parts: isolated CABG, isolated valve surgery and valve surgery plus CABG^17,19,20^. The validation database affirmed the clinical application value of this system^19^. In recent years, there were reports that STS risk evaluation system was well-validated in British, New Zealander and in Indian patients (in which it had satisfactory calibration power but poor discriminatory power) undergoing heart surgery^2,23,24^. In our study, STS risk evaluation system achieved positive calibrations (H-L: *P* > *0*.*05*) in the entire cohort and in subsets, which was in accordance with Zhang *et al*.^23^. They reported that this system might be a potentially appropriate choice for Chinese patients undergoing isolated CABG. But discrimination of STS risk evaluation system (AUC = 0.687), as well as EuroSCORE II (AUC = 0.647), was poor in high-risk group. One possible reason was that the preoperative parameters of patients in high-risk group had dramatic difference. Another possible reason was that EuroSCORE II and STS risk evaluation system also predict others cardiac surgical mortality, evaluating the predictive capacities of isolated CABG mortality may undermine its potency.

SinoSCORE solved the problem that China did not have its own heart surgery risk evaluation system. Although just started, SinoSCORE has achieved good assessments in several medical centers throughout China^25–30^. Therefore, in theory, SinoSCORE should be most relevant to Chinese patients compared with others. In our study, SinoSCORE remained the most valuable risk evaluation system (AUC = 0.888). There are several reasons. First, our study database shared the same human race with SinoSCORE database. Second, There were more similar risk factors between our study database and SinoSCORE database, such as sex, peripheral vascular disease, active endocarditis, critical preoperative state^3,4^, and which might be the reason contributed to SinoSCORE had excellent expected power. Third, all the patients in the modelling of SinoSCORE were patients only underwent CABG while patients underwent different kinds of cardiac operations were subjected to EuroSCORE II and STS risk evaluation system.

As we all know, for the risk evaluation systems, it is more meaningful to improve the ability of predicting high risk patients. Although the discrimination of the three risk evaluation systems in the high-risk group was lower than the discrimination in the low-risk group, SinoSCORE was the best discrimination in high-risk group. A part of patients in the study were involved in the establishment of SinoSCORE, which might be the reason contributed to the discrimination of SinoSCORE in high-risk group is satisfactory. Although the three systems all had good calibration and discrimination, unfortunately, they sensibly underestimated the mortality in the entire cohort and subsets. One possible reason was that although cardiac surgery and perioperative care in China have developed rapidly in the last decades, there are still some gaps compared with the developed countries. Another possible reason was that there were 3.87% of patients (65 cases) excluded from the study because of incomplete data. The discrimination of risk evaluation systems was tested by AUC, which was used to assess how well the system could discriminate between survivors and non-survivors. Therefore, AUC is considered to be one of the most important indicators to evaluate the systems. AUC is an indicator of the comprehensive evaluation system, which is more important than the predictive accuracy.

There are some limitations of the study. First, this study was a double-center retrospective and non-randomised observational study. Second, the population size was still small compared with other systems that were sourced from a large number of patients. Third, EuroSCORE II and STS risk evaluation system are designed for variety cardiac surgery, And STS risk evaluation system can also predict other outcomes. Evaluate the predictive capacities of EuroSCORE II and STS risk evaluation system to predict only isolated CABG mortality may undermine its potency. The above points might contribute to bias. Therefore, the mortality statistics maybe limited to some degree.

In summary, for isolated CABG operation in East China patients, SinoSCORE fits the data well, with excellent discrimination and good calibration. SinoSCORE showed no compromise when compared with EuroSCORE II and STS risk evaluation system.

## Methods

The study included all patients (1681 enrolled) undergoing isolated CABG in two hospitals (the first affiliated hospital of Nanjing Medical University and the east hospital affiliated to Tongji University) between January 2010 to December 2016, which was approved by ethics committees of the two hospitals. All experiments were performed in accordance with relevant guidelines and regulations. Written informed consent was obtained before data collection. There were 65 (3.87%) patients excluded from the analyses because of incomplete data, and a total of 1616 procedures comprised the study’s database. The database included 1267 males and 349 females, with an average age of 65.21 ± 8.50 years. Each patient’s diagnosis was confirmed by coronary arteriography. According to the study database, the operative risk was predicted using the algorithms online SinoSCORE available at http://www.cvs-china.com/sino.asp, EuroSCORE II available at http://www.euroscore.org/calc.html and STS risk evaluation system available at http://riskcalc.sts.org/STSWebRiskCalc273/de.aspx. The predictive mortality of each patient was ascertained by each of the systems. The definition of mortality was post-operative in-hospital death and included against-advice discharge deaths.

To further explore the predict efficacy of the three evaluation systems, in each set, it was divided into two subgroups according to the realized mortality rate (1.92%, 31/1616): high-risk group (predictive mortality ≥1.92%) and low-risk group (predictive mortality <1.92%). The calibration and discrimination of the three systems in total patients and each subset were assessed, and were compared. In order to make a fair comparison among the three systems, we compared the predictive and realized mortality rates in total and each subset.

### Statistical Analysis

The baseline data were presented as means ± standard deviation, interquartile rang for continuous variables and calculated by the t test; categorical variables were expressed as percentages and were calculated by the *χ* ^2^ (chi-square) test. *P* < *0*.*05* was considered as the statistically significant level.

Calibration and discrimination were used to assess predictive efficiency. The calibration was assessed by the Hosmer-Lemeshow (H-L) statistics. The calibration is considered to be good if *P > 0*.*05*, which indicates that the system could predict mortality accurately^31^. The discrimination was assessed by C statistics using the area under the receiver operating characteristic curve (AUC). Discrimination measured the evaluated system’s capacity to differentiate the individuals by illness or death. AUC ranges 0.50–1.00, and AUC > 0.70, > 0.75, and > 0.80 indicates that the discrimination is available, good and excellent, respectively^32^.

Calibration plots of realized versus predictive mortality rates for 20 equally sized groups by ranked predictive risk calculated of the three systems were constructed. The ideal calibrated predictions consist with the 45° line. When points below or above the diagonal indicates overestimation or underestimation respectively.

The net benefit of three risk evaluation systems for predicting in-hospital mortality was performed by Decision Curve Analysis (DCA). DCA consists in the subtraction of the proportion of all patients who are false-positive from the proportion who are true-positive, weighting by the relative harm of a false-positive and a false-negative result^33^. The statistical analysis was performed with SPSS Version 18 (SPSS Inc., Chicago, Illinois, USA). DCA was performed with R software version 3.4.0 (The R Foundation for Statistical Computing; State of Jersey, Austria) with package Decision curve.

### Data Availability

All data generated or analyzed during this study are included in this published article.
